# Examination of Mr. Samuel Young's Remarks on Mr. Astley Cooper's Operation of Tying the Aorta

**Published:** 1819-03

**Authors:** 


*For the London Medical and Physical Journal.*

### *Examination of Mr. Samuel Young's Remarks on Mr. Astley Cooper's Operation of tying the Aorta;* by C. R.

HAVING taken up the Number of the Medical Journal for March last, I was surprised at some remarks I there found, from the pen of Mr. Samuel Young, on Mr. Cooper's operation of tying the aorta. I was the more astonished at his observations, as, happening to be in town, I had the good fortune to witness an operation, which, for "delicacy and dexterity," could not be exceeded; and also to be present at the examination of the body after death. That the operation was required to save the life of the patient, Mr. Young admits; and to the manner in which it was performed he cannot raise the shadow of an objection: "but the points to which," says this gentleman, "I wish to draw the attention of your readers, is the apparent want of physiological deduction before, as well as pathological research after, the death of the patient.

As a former pupil of Mr. Astley Cooper, and one among his numerous admirers, I lament that such ill-founded accusations against a gentleman, whose unremitting zeal in his physiological and pathological pursuits is so well known to the world, should so long have remained without refutation; and shall endeavour to show that, of Mr. S. Young's charges, some are founded on an entire ignorance of, or inattention to, the circumstances of the case, and others on what appears to me to be erroneous physiology.

The first of Mr. Young's objections,—viz. "want of physiological anticipation," seems to arise from Mr. Cooper not relieving the evidently-overcharged vessels of the head by blood letting, by opening one or both jugulars. In answer to it, I shall merely request the attention of your readers to a few extracts from the "Surgical Essays," and leave them to judge whether the condition to which the patient was reduced by repeated losses of blood would have allowed one or both jugulars to have been opened, without taking from him the small chance of life which the operation afforded him. The Report says,—he was admitted into the hospital on the 9th of April; on the 16th of May, the tumor suddenly increasing in size, he was ordered to lose twelve ounces of blood from the arm; on the 21st, twelve ounces more were drawn; on the 20th of June, he had a slight bleeding from the aneurismal sac; on the 22d, the bleeding returned; on the 24th, the hæmorrhage again recurred; on the 25th, in consequence of a sudden mental agitation, he bled profusely, and, but for immediate assistance, would have expired. "At nine o'clock," says Mr. Cooper, "I found him so reduced that he could not survive another hæmorrhage; and, to prevent a further loss of blood, with which he was every moment threatened, the operation of tying the vessel was had recourse to." After the operation, the report goes on to state, that he complained of pain not only in his head, but all over his body; his pulse was weak and fluttering; his fæces and urine passed away involuntarily; his body was covered with cold sweats; together with continual vomiting.—Such is the condition of a patient in whom it is recommended, by Mr. Samuel Young, to open one or both jugulars.

Mr. Young's physiological reasoning appears as incorrect as his practice, if pursued, would have proved destructive. Surely it would have been more becoming in this gentleman to have looked to his own physiological foresight, before hastily venturing the assertion that the consequences of a ligature on the aorta would be "the retrograding the whole circulation back into the vessels of the head and chest, except such portion as would escape by collateral vessels to circulate through the lower extremities.

Before attempting to controvert this unsupported opinion as a physiological point, I will lay before your readers the examination of the body, that they may see how far appearances accord with Mr. Young's opinion. Let it be remembered that the ligature was situated three quarters of an inch above the bifurcation of the aorta, and consequently below the superior and inferior mesenteric arteries: if the whole mass of blood had been retrograded into the vessels above the ligature, surely those arteries situated *immediately* above the impediment to the progress of the blood would have been most gorged, and we ought to have found those parts supplied by the mesenteric arteries particularly tinged with blood; but the intestinal canal was not only free from inflammation or congestion, but remarkably exsangueous, as were the other abdominal viscera. The thorax I saw examined; and neither the heart nor lungs were more loaded with blood than usual. Why the head should be more likely to be incommoded with vascular determination than the thorax or abdomen, Mr. Young should have pointed out; and he would then have offered something like an argument to substantiate his assertions.

When a ligature is thrown about a vessel, is it a necessary consequence that the blood should take a retrograde course into the vessels above the obstruction to its passage, and thus produce in them a state of congestion?

Although the femoral artery be tied, the absolute quantity of blood in the limb is not diminished: it contains as much blood as before the operation; and the only difference produced in the circulation is a diminution in the rapidity of its passage through the capillaries, in consequence of its circuitous course through the anastomosing branches, and a consequent diminution in the heat of the limb. That the larger arteries of a limb, whose main trunk is tied, contain their usual quantity of blood, was shown by a case which occurred during my studies at the hospital. A man was admitted for a fungous tumor below the knee, for which the surgeon tied the femoral artery. A few days after, the tumor continuing to increase rapidly, amputation was had recourse to, and the whole of the vessels bled as freely after the operation as if the artery had not been tied. If the circulation in the arteries of the limb be rendered slower by the operation of the ligature, the return of blood in the veins must be proportionably retarded; and thus the right side of the heart will not be so freely supplied with blood, and congestion thus prevented; and, if the vessels of the limb contain as much blood as usual, what is there to produce that overcharged state of the general circulating system alluded to by Mr. Young!

Thus, then, it would appear that Mr. Young's conclusions as to the death of the patient,—viz. "that it arose from the congested state of the blood-vessels of the head, heart, and lungs, &c." are "entirely opposed to the facts of the case," and are at variance with the known effects of ligatures on arteries.

If the man's death were not occasioned by the determination of blood to the upper parts of the body, as I trust I have fully shown, we need not, surely, look for a more satisfactory explanation of the cause than that offered by the author,—viz. want of circulation in the aneurismal limb. But, says Mr. Young, no gangrene took place. I would ask, what is the distinction between a limb assuming a cold and livid state, and the incipient stage of gangrene? If by gangrene is meant putrefaction, certainly I must admit that no such process had taken place; but the contents of the aneurismal sac were in a state of putrefaction. Mr. Young must have very extraordinary notions of the animal economy, if he can suppose that nature will not take cognizance of the circulation in a whole limb being permanently impeded, and can refuse to consider such circumstances as sufficient to derange, and at length destroy, the functions of the whole machine.

The operations and discoveries of eminent men rarely meet with the approbation of cotemporaries: criticism usually falls with more severity on them, not, as Mr. Young says, because their errors are more mischievous in proportion to their eminence and utility, but because there are men who can seldom brook the superiority of others, and who feel a satisfaction in endeavouring to bring them down to their own level. Mr. Cooper's operation of tying the aorta will hereafter rank among the boldest and most important that modern surgery can boast of; and, though perhaps a few individuals may cavil, their attempts to diminish the merits of the operation must fail, from the futility of the objections that are brought forward.

*Bristol; Jan.* 18, 1819.

